# The two-stage location model for multi-construction period of material warehouses

**DOI:** 10.1371/journal.pone.0317027

**Published:** 2025-04-09

**Authors:** Wenbin Zhang, Yuejun Jin

**Affiliations:** 1 School of Mathematical Sciences, Jiangsu Second Normal University, Nanjing, China; 2 Jiangsu Anfang Electric Power Technology Co., Ltd., Taizhou, Jiangsu, China; Ataturk University, TÜRKIYE

## Abstract

This article proposes a two-stage location model to construct a unified construction plan for multi-construction period material warehouses in the distribution network. Initially, we propose a honeycomb grid partitioning method aimed at managing the time-scale of electric power material demand through effective grid administration. Subsequently, we develop an optimization model for the first stage, which identifies the threshold for the upper limit of material transportation time and establishes the construction objectives for material warehousing during each construction period. To address the challenge posed by the inconsistent locations of material warehouses across different construction periods, we formulate an optimization model for the second stage. Finally, based on the spatial distribution of power demand in Taizhou City’s distribution network, we devise a comprehensive construction plan for a material warehouse system that spans four construction periods. By adding nine material warehouse locations, we achieve a reduction of 57.14% in the maximum transportation time for electric power materials. The method proposed in this article provides a general solution for the long-term planning of persistent structures by converting long-term planning into construction goals across multiple stages.

## 1. Introduction

The direct power supply from the distribution network to users is one of the critical infrastructures of society [1]. Service interruptions in the distribution system not only lead to financial losses for power companies but also have serious repercussions for society [2]. Improving the timeliness of material delivery is an effective measure to reduce the duration of power outages and mitigate losses [3,4]. Therefore, the location problem of material warehouses is an important research focus in the construction of the distribution network.[5].

Existing research on the location problem of material warehouses mainly concentrates on two aspects. One is multi-criteria decision-making. There is a wealth of re-search results in this area, with the most common method being the Analytical Hierarchy Process (AHP), which primarily relies on pairwise comparisons of the importance of factors [6].To further consider the interrelations among factors, the Analytic Network Process (ANP) has been proposed [7,8].To fully utilize the information in the original data and accurately reflect the differences among various evaluation methods, the fuzzy Order Preference Similarity to the Ideal Solution (TOPSIS) has been constructed [9]. Additionally, the Vlsekriterijumska optimizacija I kompromisno resenje (VIKOR) method has been introduced to eliminate the situation where individual negative indicators are easily overlooked [10]. Recently, a fuzzy multi-attribute and multi-actor decision-making (FMAADM) approach has also been proposed [11].

Another aspect is the mathematical model. Based on the demand situation, existing mathematical models can be classified into two categories. One category pertains to cases with deterministic demand. Research findings in this area include single-objective optimization models focusing on time and cost [12,13], as well as multi-objective optimization models that consider multiple factors such as economic, technical, social, and safety aspects [14–16]. The other category deals with scenarios involving uncertain demand. Research outcomes in this domain encompass stochastic optimization models [17,18], fuzzy optimization models [19,20], and robust optimization models [21,22].

Despite the existence of numerous studies addressing the location of material warehouses, the majority of these investigations focus on short-term, single optimization models. There is a paucity of literature concerning the long-term planning of persistent structures, particularly with respect to the simultaneous consideration of multi-period optimization. The construction cycle for material warehouses within distribution networks is notably lengthy and frequently requires segmentation into multiple construction phases to guarantee the timely provision of materials. However, under varying transportation time constraints, the optimal locations for material warehouses, as determined by multi-criteria decision-making and mathematical models, often yield inconsistent results. In essence, the prevailing methodologies and models are inadequate for addressing location problems that necessitate completion across multiple construction phases.

**Fig 1:**
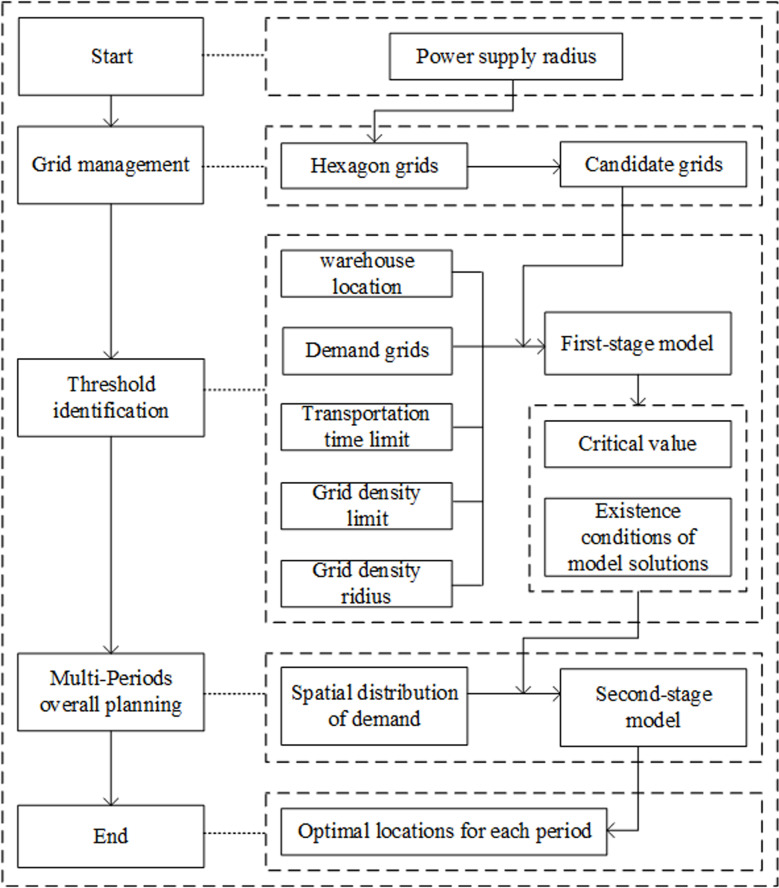
Flow chart of the two-stage location model for multi-construction period of material warehouses.

In order to fill the aforementioned gap, this article will propose a two-stage location model to establish a construction plan for material warehouses over multiple construction periods in the distribution network. The flow of this paper (See Fig 1) is as follows:

The remainder of this paper is organized as follows. Section 2 presents the symbols and model parameters. Section 3 establishes the two-stage location model for multi-construction period of material warehouses. Section 4 completes empirical analysis. Section 5 presents the conclusion.

## 2. Symbols and model parameters

### 2.1. Symbols

The symbols used in this paper are shown in Table 1.

**Table 1:** The symbols.

$r_{hexagon}$	Radius of the hexagonal grid
$x_{i}$	Location of electric power material demand, i=1,⋯,N
$X_{i}$	Hexagonal grid where the demand point $x_{i}$ is located, i=1,⋯,n
${\hat{X}}_{j}$	Hexagonal grid for candidate warehouse location, j=1,⋯,m
$d_{max}$	Transportation time limit for electric power material
$d_{ij}$	Distance from demand grid $X_{i}$ to candidate grid ${\hat{X}}_{j}$
${\hat{d}}_{ij}$	Distance from candidate grid ${\hat{X}}_{i}$ to candidate grid ${\hat{X}}_{j}$
$f\left(d_{ij}\right)$	Service quality function of candidate grid ${\hat{X}}_{j}$ on demand point $x_{i}$
$a_{ij}$	Path parameters from candidate grid ${\hat{X}}_{j}$ to demand grid $X_{i}$
$R_{h}$	Grid density radius
$H_{\max}$	Grid density limit
$T_{\max}$	Transportation time limit
$P_{k}$	Construction targets for the of the $k_{th}$ warehouse

### 2.2. Honeycomb grids

**Fig 2:**
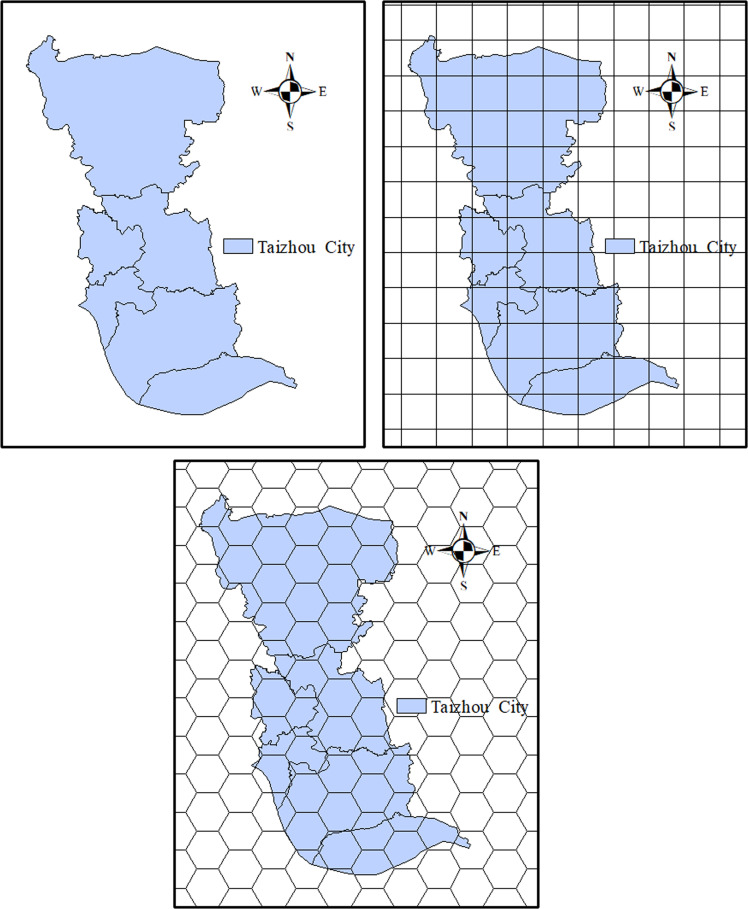
Regular polygon grid: (a) Administrative region; (b) Square; (c) Hexagon.

Discretizing the coverage area of the power grid into different grid regions is a common method for addressing the location problem of material warehouses. However, the existing administrative districts exhibit inconsistencies in their shapes and sizes (Fig 2 (a)) [23], and the distances between adjacent square regions are not uniform (Figure 2 (b)) [24], making it difficult to meet the precise planning requirements at a fine time scale.

To address this issue, we will implement a hexagonal grid [25], as it is the geometric shape that most closely approximates a circle and exhibits the highest level of consistency among various grid configurations (see Fig 2(c)). This approach facilitates the grid-based management of electric power material demand. Meanwhile, we set the radius of the hexagonal grid as $r_{hexagon}$.

### 2.3. Service quality function

Transportation time is a key factor in determining the service quality of material warehouse for material demand points. We assume that the service quality function $f\left(d_{ij}\right)$ of candidate grid $X_{j}$(j=1,⋯,m) for demand grid (i=1,⋯,n) is as follows:

$$f\left(d_{ij}\right)\text{=} \{\begin{array}{c} \begin{array}{cc} 1, & \;\;\;d_{ij}\leq T_{0}*v_{ij} \end{array}\\ \begin{array}{cc} \frac{d_{ij}-T_{0}*v_{ij}}{T_{max}*v_{ij}-T_{0}*v_{ij}}, & T_{0}*v_{ij}<d_{ij}\leq T_{max}*v_{ij} \end{array}\\ \begin{array}{cc} 0, & \;\;\;d_{ij}>T_{max}*v_{ij} \end{array} \end{array},$$ (1)

where $T_{0}$ represents the optimal transportation duration for electric power materials, $T_{max}$ denotes the upper limit of transportation time, and $v_{ij}$ indicates the driving speed of the vehicle transporting from the demand point $x_{i}$ to the candidate grid, ${\hat{X}}_{j}$, i=1,⋯,N, j=1,⋯,m.

### 2.4. Spatial distribution density constraint function

It is noted that the denser the construction of material warehouses, the higher the required construction costs. To alleviate the financial pressure caused by excessive density of material warehouse construction, we use the frequency of material warehouses within a circular area of radius $R_{{}_{h}}$ to limit the construction density of these facilities. Thus, we establish the following grid density constraint function $h\left({\hat{X}}_{j}\right)$ at the deployment point ${\hat{X}}_{j}$:

$$h\left({\hat{X}}_{j}\right)\text{=}\sum_{k=1}^{M}g\left({\hat{d}}_{jk}\right){\hat{X}}_{j}{\hat{X}}_{k},$$ (2)

$$g\left({\hat{d}}_{jk}\right)\text{=} \{\begin{array}{l} 1,\;{\hat{d}}_{jk}\leq R_{h}\\ 0,\;{\hat{d}}_{jk}>R_{h} \end{array}.$$ (3)

Here, ${\hat{d}}_{jk}$ represents the Euclidean distance between the center point of grid ${\hat{X}}_{j}$ and the center point of grid ${\hat{X}}_{j}$, j,k=1,⋯,m. Meanwhile, we refer to $R_{{}_{h}}$ as the grid density radius, and $H_{\max}$ as the maximum value of $h\left({\hat{X}}_{j}\right)$, which serves as the upper limit for grid density.

## 3. Model construction

Based on the idea of transforming long-term construction planning issues into multiple construction phases, we will construct a two-stage location-allocation model that covers the entire material demand of the distribution network, aimed at determining the objectives and spatial locations for all material warehouse constructions over multiple construction periods. The first stage focuses on identifying the objectives for material warehouse construction during different construction periods and analyzing the threshold for transport time limits, as well as the conditions for the optimal solution to exist in the allocation model. The second stage involves establishing a coordinated planning model for the construction objectives across different periods, ensuring spatial consistency in the material warehouse construction objectives during various timeframes, and determining the objectives and locations for material warehouse construction in each construction period.

### 3.1. Single-period construction objective identification model

It is noted that in the site selection process for material storage, we need to prioritize time to ensure that electrical materials can be delivered to the demand locations as quickly as possible. In other words, the construction of material warehouse is non-economic in nature and belongs to an optimization model aimed at meeting full demand coverage.

Considering that the construction objectives for material warehouse are influenced by factors such as transport time limits, grid density radius, grid density upper limit, and construction costs, we construct an optimization model in the first stage with the goal of minimizing construction costs as the objective function. Through evolutionary analysis, we identify the construction objectives for material warehouse and analyze the conditions for the existence of solutions to the model.

The model for the first stage is as follows:

$$Min\;F=\sum_{i=1}^{n}c_{j}{\hat{X}}_{j}.$$ (4)

Constraint conditions:

$$\sum_{j=1}^{m}a_{ij}x_{i}\geq 1,i=1,\cdots ,n,$$ (5)

$$a_{ij}\leq {\hat{X}}_{j},$$ (6)

$$d_{ij}a_{ij}\leq T_{max}*v_{ij},i=1,\cdots ,n,j=1,\cdots ,m,$$ (7)

$$h\left({\hat{X}}_{j}\right)\leq h_{\max},j=1,\cdots ,m,$$ (8)

$${\hat{X}}_{j}\in \left\{0,1\right\}$$ (9)

$$a_{ij}\in \left\{0,1\right\},$$ (10)

The objective function (4) represents the minimization of construction costs for material storage, where $c_{j}$ denotes the cost weight for establishing material warehouse at grid ${\hat{X}}_{j}$. Constraint (5) indicates that the electrical material demand at demand point $x_{i}$ is covered; constraint (6) ensures that the coverage route for the electrical material demand at demand point $x_{i}$ is meaningful; constraint (7) states that the electrical material demand at demand point $x_{i}$ must be delivered within the specified time; constraint (8) sets the upper limit on the grid density for the spatial distribution of material storage; constraints (9) and (10) specify that $a_{ij}$ and ${\hat{X}}_{j}$ are integers that can only take values of 0 or 1.

### 3.2. Multi-Construction Period Location Model

The construction period for material warehouses is relatively long, and the goal of timely delivery of materials must be achieved gradually through multiple construction periods. However, the construction objectives for material warehouse under different thresholds not only differ in quantity but also exhibit inconsistencies in spatial location. To address this, we propose a multi-construction period location model to ensure consistency in the locations of material warehouses across different construction periods.

Let ${\left\{T_{k,max}\right\}}_{k=1}^{K}$ be the threshold for transport time limits obtained from Model (4), ${\left\{P_{k}\right\}}_{k=1}^{K}$be the threshold for the frequency of material warehouse construction objectives,and ${\left\{H_{k,max}\right\}}_{k=1}^{K}$ , ${\left\{R_{k,h}\right\}}_{k=1}^{K}$ represent the upper limit of grid density and grid density radius,respectively, for the existence of the optimal solution in Model (4).

The optimization model for the second stage is as follows:

$$Max\;F=\sum_{i=1}^{n}\sum_{j_{k}=1}^{m}\sum_{k=1}^{l}f\left(d_{ij_{k}}\right){\hat{X}}_{j_{k}}$$ (11)

Constraint conditions:

$$a_{ij_{k}}\leq {\hat{X}}_{j_{k}},i=1,\cdots ,n,j_{k}=1,\cdots ,m,\forall k,$$ (12)

$$\sum_{j_{k}=1}^{m}a_{ij_{k}}x_{i}\geq 1,j_{k}=1,\cdots ,m,\forall k,\forall i,$$ (13)

$$d_{ij_{k}}a_{ij_{k}}\leq T_{k,max}*v_{ij_{k}},i=1,\cdots ,n,j_{k}=1,\cdots ,m,\forall k,$$ (14)

$$\sum_{j_{k}}{\hat{X}}_{j_{k}}=P_{k},\forall k,$$ (15)

$${\hat{X}}_{j_{k}}\leq {\hat{X}}_{j_{k+1}},\forall k,$$ (16)

$${\hat{X}}_{{j^{\prime }}_{k}}=1,{j^{\prime }}_{k}\in A,\forall k,$$ (17)

$$h\left({\hat{X}}_{j_{k}}\right)\leq h_{\max},j=1,\cdots ,m,$$ (18)

$$a_{ij_{k}}\in \left\{0,1\right\},$$ (19)

$$X_{j_{k}}\in \left\{0,1\right\}.$$ (20)

The objective function (11) aims to maximize the service quality of material warehouse across all periods. Constraint (12) indicates that the coverage routes for the electrical material demand at the demand point $x_{i}$ during all periods must be meaningful; constraint (13) ensures that the electrical material demand at the demand point $x_{i}$ during different periods is fully covered; constraint (14) states that the electrical material demand at the demand point $x_{i}$ during different periods must be delivered within the specified time; constraint (15) specifies the construction quantity of material warehouses for the K-th period; constraint (16) stipulates that the construction objectives for material warehouse in the K-th period are included within the construction objectives for the subsequent period; constraint (17) requires that the existing material warehouses remain unchanged across different construction periods; constraint (18) ensures that the spatial distribution of material warehouse meets the upper limit for grid density; constraints (19) and (20) specify that $a_{ij_{k}}$ and ${\hat{X}}_{j_{k}}$ are integers that can only take values of 0 or 1.

## 4. Empirical analysis

Based on the empirical observation that the power supply radius of medium voltage lines in urban areas does not exceed 3000 meters, we set the radius $r_{hexagon}$ of the honeycomb grid to 2000 meters, dividing Taizhou City into 2704 honeycomb grids. At the same time, we take the average driving speed of trucks on provincial roads to be 40 kilometers per hour, which serves as the vehicle speed for short time scales. To address the challenge of integer optimization models with a large number of variables, this paper employs a method that integrates MATLAB and Gurobi to solve Models 4 and 5.

### 4.1. Threshold identification

**Fig 3:**
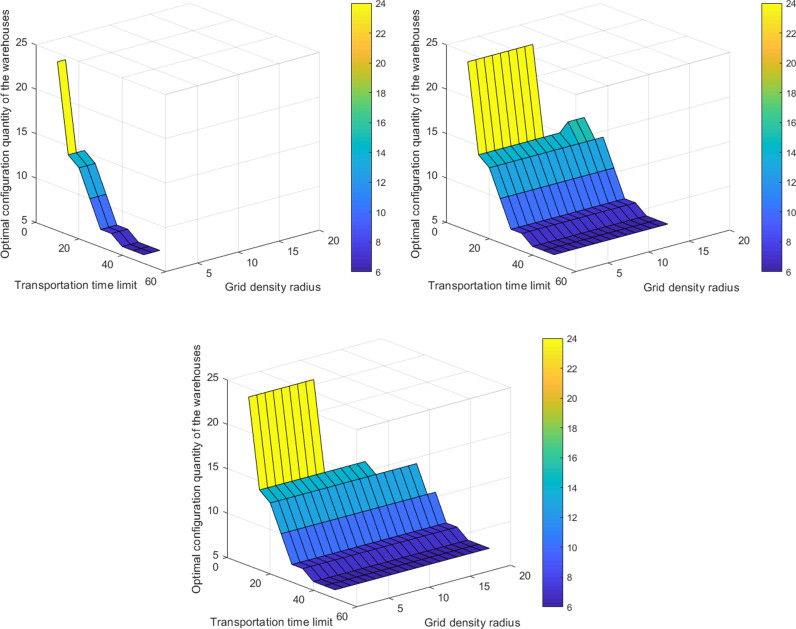
The three-dimensional coupling evolutionary relationship between the optimal configuration quantity of material storage, transport time limits, and grid density radius: **(a)** $H_{\max}\text{=}1$; **(b)** $H_{\max}\text{=}2$; **(c)** $H_{\max}\geq 3$.

According to Model [4], we set the upper limit for grid density $H_{\max}$ to be: 1, 2, 3, 4, 5, 6, and obtain the optimal configuration quantity of material warehouse along with the three-dimensional coupling evolutionary relationship between transport time limits and grid density radius (Fig 3).

**Fig 4:**
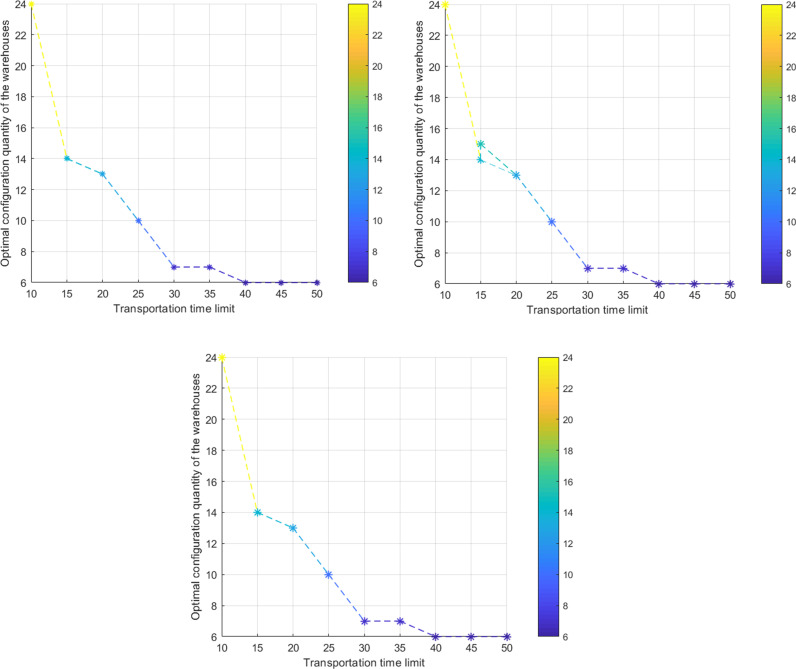
The two-dimensional coupling evolutionary relationship between the number of new material warehouses *α* and the transport time limit $T_{max}$: **(a)** $H_{\max}\text{=}1$; **(b)** $H_{\max}\text{=}2$; **(c)** $H_{\max}\geq 3$.

There is a noticeable inverse relationship between the transport time limits $T_{max}$ and the number of new material warehouses *α* (Fig 3). When the maximum value $T_{max}$ is set at 75 minutes, the existing material warehouses satisfies the system demand, and *α* reaches the minimum value of 0; when the minimum value $T_{max}$ is set at 15 minutes, the number of new material warehouses *α* reaches a maximum value of 18. Throughout the process of gradually decreasing the value of $T_{max}$ from 75 to 52.5, to 37.5, to 30, to 22.5, and finally to 15, the value of *α* gradually increases from 0 to 1, then 4, 7, 8, and ultimately 18. Moreover, this inverse relationship remains unchanged regardless of variations in the value of $H_{\max}$ (Figs 4 (a)-(c)).

Remark: When the maximum transportation time $T_{k,\max}$ is less than 15 minutes, the spatial distance from the material warehouses to the demand points does not exceed 10 kilometers. Additionally, when the number of newly added material warehouses exceeds 18, the construction cost becomes excessively high and lacks practical significance. Therefore, scenarios with values of $T_{k,\max}$ less than 15 have not been discussed.

**Fig 5:**
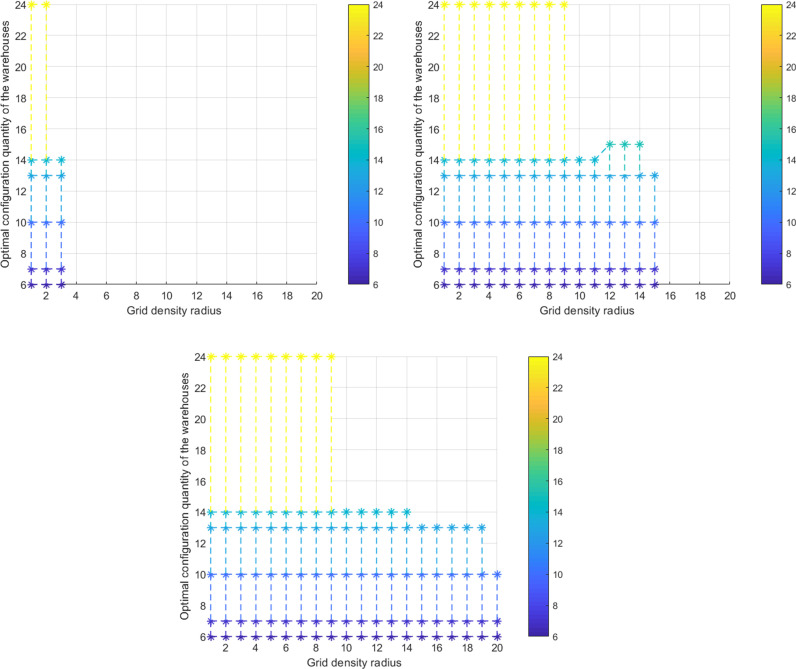
The two-dimensional coupling evolution relationship between the number of new material warehouses *α* and the grid density radius $R_{{}_{h}}$: **(a)** $H_{\max}\text{=}1$; **(b)** $H_{\max}\text{=}2$; **(c)** $H_{\max}\geq 3$.

The impact of grid density radius $R_{{}_{h}}$ and grid density limit $H_{\max}$ on the number of new material warehouses *α* is not significant. When *α* takes a specific value of 0 (1, 4, 7, 8), $R_{{}_{h}}$ takes the values: 1,2,3 ($H_{\max}\text{=}1$, Figure 5(a)); 1,2,…,15($H_{\max}\text{=}2$, Fig 5(b)); 1,2,…,20($H_{\max}\geq 3$, Fig 5(c)).

**Fig 6:**
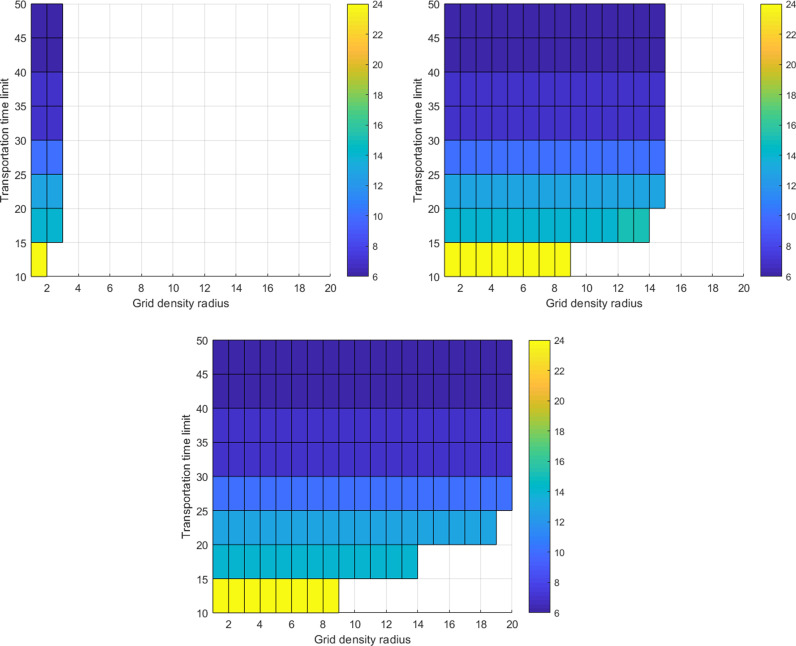
The two-dimensional coupling evolutionary relationship between transport time limits $T_{max}$ and grid density radius. $R_{{}_{h}}$: **(a)** $H_{\max}\text{=}1$; **(b)** $H_{\max}\text{=}2$; **(c)** $H_{\max}\geq 3$.

The upper limit of grid density $H_{\max}$ for material warehouse has a significant impact on the existence of solutions to Model (4). As the value of $H_{\max}$ increases, the blank areas representing scenarios where the model has no solution noticeably decrease. Furthermore, when the value of $H_{\max}$ reaches 3, the blank area indicating no solution reaches its minimum and remains unchanged (Fig 6).

When the upper limit of grid density $H_{\max}$ is fixed at 1, we statistically analyze the numbers of new material warehouse as the transport time limit $T_{max}$ varies (Table 2). We find that there is no intersection among the sets of grid numbers corresponding to different values of $H_{\max}$.

**Table 2:** The grid numbers of new added material warehouse with varying values of $T_{k,max}$ and $H_{\max}$.

	$T_{k,max}$				
	$\left(45,52.5\right]$	$\left(37.5,45\right]$	$\left(30,37.5\right]$	$\left(22.5,30\right]$	$\left(15,22.5\right]$
$H_{\max}\text{=}1$	2702	2539	917,1356, 2075, 2184	535,686,1177, 1239,1561, 2198,2265	269,343,757, 779,1350,1849, 2267,2645
$H_{\max}\text{=}2$	2457	2312	744,927, 1545, 2361	341,623,842, 1543,2123, 2268,2691	269,440,740, 908,1350,1849, 2282,2660
$H_{\max}\geq 3$	2457	2312	744,927, 1545, 2361	341,623,842, 1543,2123, 2268,2691	8,381,582, 779,1350,1849, 2315,2660

Although the number of new added warehouses *α* is 1 when $T_{max}$ belongs to $\left(45,52.5\right]$ and $\left(37.5,45\right]$, the respective grid numbers are still different, namely 2702 and 2539. Next, when the upper limit of the grid density function $H_{\max}$ is set to 2 and 3, we again analyze the numbers of new material warehouses as the transport time limit $T_{max}$ changes (Table 2) and arrive at similar conclusions.

The optimal solution of Model 4 varies with parameters $T_{k,max}$ and $H_{\max}$. When $T_{k,max}$ falls within interval $\left(45,\;52.5\right]$, the number of new added warehouses *α* for Model 4 is one, and the spatial position of these new added warehouses remains constant as long as the value of parameter $H_{\max}$ is specified. Specifically, when $H_{\max}\text{=}1$, the grid position of the new added warehouse is 2702; when $H_{\max}\text{=}2$, the grid position is 2457; and when $H_{\max}\geq 3$, the grid position is also 2457.

When $T_{k,max}$ belongs to interval $\left(37.5,\;45\right]$, the number of new added warehouses for Model 4 remains one, and the spatial position of the new added warehouse remains unchanged as long as the value of parameter $H_{\max}$ is specified. Specifically, when $H_{\max}\text{=}1$, the grid position of the new added warehouses is 2539; when $H_{\max}\text{=}2$, the grid position is 2457; and when $H_{\max}\geq 3$, the grid position is also 2457. When $T_{k,max}$ belongs to other intervals, the grid position of the new added warehouses will also remain unchanged as long as the value of parameter $H_{\max}$ is fixed (Table 2).

In summary, the transport time limit $T_{max}$ is the primary influencing factor for the number of new material warehouses *α*, with threshold values ${\left\{T_{k,max}\right\}}_{k=1}^{K}$ of 52.5, 37.5, 30, 22.5, and 15 minutes, corresponding to material warehouse construction objective frequencies ${\left\{P_{k}\right\}}_{k=1}^{K}$ of 1, 4, 7, 8, and 18, respectively. Additionally, the spatial locations of material warehouses under Model (4) during different construction periods lack consistency, which does not meet the requirements for a unified construction plan for multi-construction period material warehouses.

### 4.2. An overall warehouse construction scheme with multi-period

Based on the research results described above, we set the grid density radius $R_{{}_{h}}$ to be 5 kilometers and the upper limit of grid density $H_{\max}$ to be 3. In order to achieve unified planning for the locations of material warehouses across various construction periods, this section compares the iterative approach of Model (4) with Model (5) and selects the optimal strategy.

First, we use the iterative approach of Model (4) while keeping the existing material warehouse locations unchanged. We calculate the locations of new material warehouses when $T_{max}$ is set to 52.5 minutes and add them to the set of existing material warehouse locations. Next, we use the updated set of existing material warehouse locations to calculate the locations of new material warehouses when $T_{max}$ is set to 37.5 minutes and add these to the set of existing locations. This process continues iteratively until we calculate the locations of new material warehouses when $T_{max}$ is set to 15 minutes using Model (4). Meanwhile, we also calculate the spatial locations of new material warehouses for the four construction periods based on Model (5).

**Fig 7:**
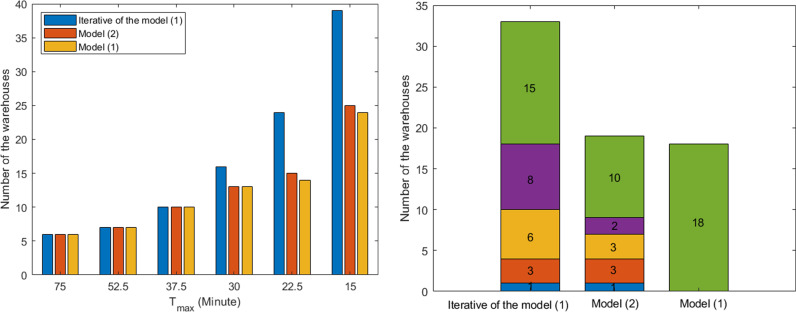
The frequency of warehouse construction under three schemes: **(a)** the frequency of warehouse construction in each stage; **(b)** Number of newly added warehouse *α* at each stage.

By analyzing the frequencies of material warehouses at each stage under the two plans (Fig 7 (a)) and the corresponding numbers of new material warehouses (Fig 7 (b)), we find that Model (5) maintains a high degree of consistency with the single optimal solution of Model (4) while ensuring that the construction locations of material warehouses at each stage are uniform. The site frequency ratios are as follows: 100%, 100%, 100%, 107%, and 104%. In contrast, under the iterative approach of Model (4), the stage construction objectives for material warehouse gradually deviate from the single optimal solution of Model (4) as $T_{max}$ decreases, with site frequency ratios of 100%, 100%, 123%, 171%, and 162% (Fig 7(a)).

Regarding the reduction of time $T_{max}$ for the number of new material warehouses *α*, the average reduction in $T_{max}$ for each site during each construction period in Model (5) is as follows: 15, 2.5, 2.5, 3.75, and 0.75 (minutes). Therefore, 22.5 minutes is identified as the optimal $T_{max}$ construction target.

**Fig 8:**
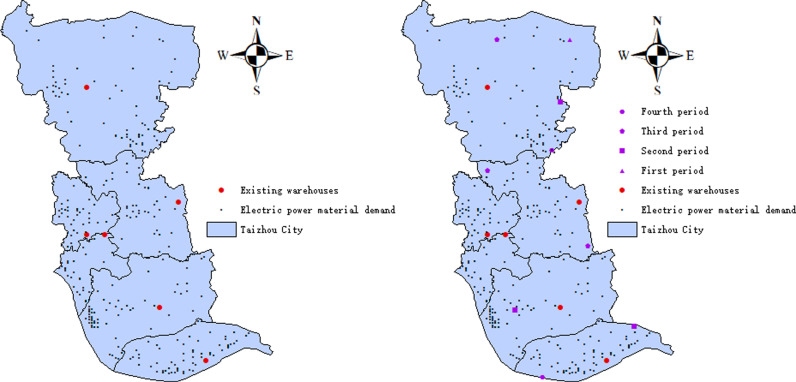
Spatial distribution of the electric material warehouses: **(a)** Existing spatial distribution; **(b)** Spatial distribution after construction.

According to Model (5), we have developed a construction plan for electrical material warehouses in the Taizhou area, which is divided into four construction periods. The spatial locations of these facilities are shown in Fig 8(b). Based on this construction plan, the number of material warehouses in Taizhou City increased from the existing 6 to 15: 6 → 7 → 10 → 13 → 15 (Fig 8(a)). Additionally, the transport time for electrical materials was reduced by 57.14%: from 52.5 minutes to 37.5, then to 30, 22.5, and finally 15 minutes.

## 5. Conclusions

The long-term planning of durable buildings is an important issue in location problem. To solve this problem, this article proposes a two-stage material warehouse location model and performs an empirical analysis utilizing data from distribution networks.

First, to ensure the timely delivery of material demands for the distribution network, we proposed a grid management system using a honeycomb grid. Next, we developed an optimization model for the first stage and clarified the relationship between the optimal configuration quantity of material warehouses, transport time limits, the radius of the grid density function, and its upper limit through evolutionary analysis, resulting in the identification of threshold values for transport time limits and objectives for material warehouse construction in each construction period. Among them, the thresholds for A in the four stages are 37.5, 30, 22.5, and 15, respectively. The target number of new storage points for each period is 1, 4, 7, and 8, respectively. However, the spatial locations of these construction targets are inconsistent.

Then, to overcome the inconsistency in the spatial locations of material warehouse construction across different construction periods, we built an optimization model for the second stage to achieve uniformity in the spatial locations of material warehouses during multi-construction periods.

Finally, based on the spatial distribution of electrical demand in Taizhou City, we established a unified construction plan that includes four construction periods: The value range of $T_{k,max}$ in the first construction is $\left(37.5,52.5\right]$, with an optimal storage grid number of 2,075. In the second construction, the value range of $T_{k,max}$ is $\left(30,37.5\right]$, and the optimal storage grid numbers are 535,907 and 248. The value range of $T_{k,max}$ in the third construction is $\left(22.5,30\right]$, with optimal storage grid numbers of 414, 1607, and 2346. Finally, the value range of $T_{k,max}$ in the fourth construction is $\left(15,22.5\right]$, with optimal storage grid numbers of 1381 and 1850. By adding 9 material warehouses, we reduced the maximum transport time for electrical materials by 57.14%.

The methodology presented in this article represents a universal approach that is applicable to the long-term planning problem of durable buildings. It only requires transforming the long-term plan into several phased construction objectives.
